# Attenuated Structural Transformation of Indaconitine during Sand Frying Process and Anti-Arrhythmic Effects of Its Transformed Products

**DOI:** 10.1155/2022/8606459

**Published:** 2022-02-17

**Authors:** Yan Wang, Pei Tao, Yu-Jie Wang

**Affiliations:** ^1^School of Pharmacy, Chengdu University of Traditional Chinese Medicine, Chengdu 611137, China; ^2^School of Ethnic Medicine, Chengdu University of Traditional Chinese Medicine, Chengdu 611137, China

## Abstract

The transformation pathways of diterpenoid alkaloids have been clarified clearly in the boiling and steaming process, but remain to be determined in the sand frying process. The aims of the study were to investigate the transformation pathways of indaconitine in the sand frying process, as well as examine the cardiotoxicity and anti-arrhythmic activity of indaconitine and its transformed products. The transformed product was separated by column chromatography, and the structure was identified by ^1^H NMR, ^13^C NMR, and HR-ESI-MS. The cardiotoxicity of indaconitine and its transformed products was clarified by observing the electrocardiogram (ECG) changes at the same dose. Furthermore, the anti-arrhythmic activity of the transformed products was investigated using an aconitine-induced rat arrhythmia model. Consequently, Δ^15(16)^-16-demethoxyindaconitine, a new diterpenoid alkaloid, was isolated from processed indaconitine. Intravenous injection of 0.06 mg/kg indaconitine induced arrhythmias in SD rats, while Δ^15(16)^-16-demethoxyindaconitine did not exhibit arrhythmias at the same dose. In the anti-arrhythmic assay, mithaconitine, obtained in the previous research, together with Δ^15(16)^-16-demethoxyindaconitine, could dose-dependently delay the onset time of ventricular premature beat (VPB) and reduce the incidence of ventricular tachycardia (VT), combined with the increasing arrhythmia inhibition rate, exhibiting strong anti-arrhythmic activities. These results indicated that two or more pathways exist in the sand frying process, and the transformed products exhibited lower cardiotoxicity and strong anti-arrhythmic activities, which had the possibility of being developed into anti-arrhythmic drugs.

## 1. Introduction

*Aconitum* L. (Ranunculaceae) is an indispensable herbal medicinal genus. There are over 200 species in China, mainly distributed in western Sichuan, northwest Yunnan, and eastern Tibet Autonomous Region, among which 76 species can be used medicinally [1, 2]. For example, *Aconitum carmichaelii* Debx., *A. kusnezoffii* Reichb., *A. pendulum* Busch, *A. tatsienense* Finet & Gagnep., and *A. kongboense* Lauener are commonly used in traditional and folk medicine in China [3–5]. These herbs are effective in the treatment of chronic pain, arthralgia, and other ailments caused by rheumatoid arthritis [6, 7].

As is well known, the unprocessed *Aconitum* herbs mainly contain toxic diterpenoid alkaloids [3–5, 8], to ensure safety and effectiveness, and they must be processed before clinical medication. Generally, *Aconitum* plants are processed by “boiling with water for 4–6 hours” or “steaming for 6–8 hours” in traditional Chinese medicine [5, 9], which has been documented in the Chinese Pharmacopoeia 2020. While the sand frying method is usually applied in Qiang and Tibetan medicine, it only takes 10–15 minutes of stir-frying with sand to achieve the purpose of detoxification. During the boiling and steaming process, the structural transformation pathways of diterpenoid alkaloids are relatively conservative, mainly adhering to the following processing principles: the acetoxyl group at C-8 and benzoyl group at C-14 of toxic diterpenoid alkaloids are unstable, easily hydrolyzed in the presence of water, and then converted to the low toxic monoester- and amine-diterpenoid alkaloids [9, 10]. For example, the acetoxyl group at C-8 of aconitine is hydrolyzed to obtain benzoylaconine, and the benzoyl group at C-14 of benzoylaconine is further hydrolyzed to generate aconine [9, 10]. However, the structural transformation pathways of these components are more complicated during the process of sand frying, and the transformation mechanisms have not been fully elucidated [11].

Mithaconitine, a sand-fried product of indaconitine, has been isolated from processed indaconitine in our previous study [12]. It was also confirmed that the cardiotoxicity was lower than that of the prototype compound indaconitine, which was the main ingredient of *A. tatsienense* Finet & Gagnep. [12, 13]. In this study, another main transformed compound was further separated and identified. Besides, the cardiotoxicity was also investigated. By comparing the changes in structure and toxicity between the prototype compound and the converted products, the structural transformation pathways of indaconitine would be clarified, as well as whether the sand frying method could attenuate the toxicity.

Notably, some diterpenoid alkaloids with similar structures exert opposite effects. For example, aconitine, a famous diterpenoid alkaloid, has fatal arrhythmogenic effects, while its structurally similar alkaloids 14-benzoyltalatisamine, 14-benzoyldelcosine, and 14-benzoylidocarpine [14–16] have anti-arrhythmic effects. Since the structure of indaconitine is similar to its transformed products, it is unclear, with the transformation of structure, whether the transformed components will generate an anti-arrhythmic effect on the premise of reduced toxicity. Therefore, the anti-arrhythmic activity of transformed components was further investigated by employing a rat model of aconitine-induced arrhythmia.

## 2. Materials and Methods

### 2.1. General Experimental Procedures

BL-420F organism functional experimental system (Chengdu Techman Co., Ltd., Chengdu, China) was used to record the lead II electrocardiograms (ECGs) of rats. CPA2250 electronic analytical balance (Sartorius, Germany) was used to correctly weigh the laboratory samples. HH-SJ heat-collecting magnetic stirrer (Jintan Chengdong Xinrui Instrument Factory, Changzhou, China) was used for simulating the process of sand frying by oil bath heating. Optical rotations were obtained on a PerkinElmer 341 polarimeter (PerkinElmer, USA). The melting point was measured on an X-4 micro-melting point apparatus and uncorrected (Shanghai Precision Scientific Instrument Corporation, Shanghai, China). NMR spectra were measured on a Bruker Avance 600 spectrometer (Bruker, Germany) in CDCl_3_ with tetramethylsilane (TMS) as an internal standard. Mass spectra were carried out on a micrO-TOF-Q-II mass spectrometer (Bruker, Germany). RE-3000B rotary evaporator (Shanghai Yarong Biochemical Instrument Factory, Shanghai, China) was used to evaporate the solvent. Silica gel G (200–300 mesh) for column chromatography and TLC plates (silica gel G) were obtained from Qingdao Sea Chemical Factory (Qingdao, China). Spots on TLC plates were visualized with Dragendorff's reagent.

### 2.2. Chemicals and Reagents

Indaconitine (purity ≥98%) and aconitine (purity = 99%) were purchased from Shaanxi Herbchem Biotechnology Co., Ltd. (Xi'an, China). Propafenone hydrochloride (purity = 99.8%) and lidocaine hydrochloride (purity = 93.4%) were purchased from National Institutes for Food and Drug Control (Beijing, China) and urethane from Chengdu Kelong Chemical Reagent Factory (Chengdu, China). Mithaconitine and Δ^15(16)^-16-demethoxyindaconitine (purity >95%) were separated and identified from processed indaconitine, and the structures were verified by ^1^H NMR, ^13^C NMR, and HR-ESI-MS. All of the reagents were analytical grade.

### 2.3. Animals

SPF-grade Sprague Dawley (SD) rats with either sex weighing between 180 g and 220 g (Certificate No. SCXK (CHUAN) 2015–030) were supplied from Chengdu Dossy Experimental Animals Co., Ltd. (Chengdu, China) and maintained under a controlled light/dark cycle and temperature (20 ± 2°C), with free access to food and water. They were left for 2 d for acclimatization to animal room conditions. Animal experiments were performed in adherence with the Guiding Principles for the Care and Use of Laboratory Animals of China and were approved by the Animal Experimentation Ethics Committee of Chengdu University of Traditional Chinese Medicine (Permission No. 2020–15).

### 2.4. Preparation and Separation of Transformed Component

520 mg indaconitine was dissolved in a 250 mL round-bottomed flask with a proper amount of dichloromethane, and the solvent was removed in vacuo to make it uniformly adhere to the inner wall of the flask. The flask was subsequently immersed in an oil bath, heated at 160°C for 30 min, and cooled to room temperature after reaction, and a crude residue of indaconitine (450 mg) was obtained for column chromatography.

The residue was subjected to column chromatography (silica gel, 120 g, 200–300 mesh) and eluted with petroleum ether-acetone-triethylamine (8 : 1:0.01 to 3 : 1:0.01) to obtain three fractions (A-C). Fraction C (100 mg) was further purified over column chromatography (silica gel, 120 g, 200–300 mesh) and eluted with petroleum ether-acetone-triethylamine 3 : 1:0.01 to afford compound **1** (50 mg).

### 2.5. Electrocardiography

The rats were anesthetized by intraperitoneal (*i.p.*) injection of 20% urethane (1.2 g/kg), with their back fixed, and four limbs in subcutaneous penetration of needle electrodes. Lead II ECGs were recorded after the administration of urethane. ECG acquisition was performed using the BL-420F organism functional experimental system.

### 2.6. Cardiotoxicity Test

**Figure 1:**
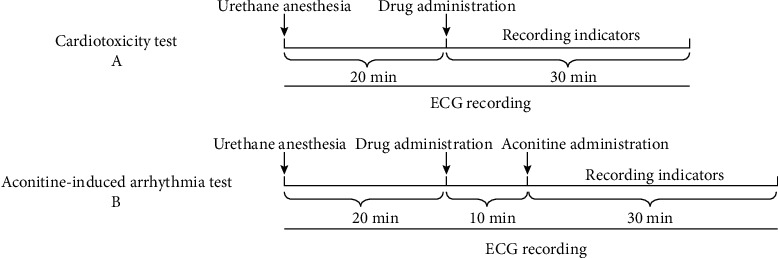
Experimental timeline.

20 male and female SD rats were randomly divided into two groups (*n* = 10 for each group): indaconitine and Δ^15(16)^-16-demethoxyindaconitine (compound **1**). At the beginning of the experiments, both groups of rats were anesthetized using 20% urethane (1.2 g/kg, *i.p.*), recording the lead II ECGs for 20 min prior to drug administration, and then, the rats were intravenously injected the same dose of experimental compounds. ECGs were continually conducted for 30 min after drug administration [12](Figure 1).

Through the preliminary experiment, it was found that 0.06 mg/kg indaconitine caused ventricular premature beat (VPB), ventricular tachycardia (VT), and ventricular fibrillation (VF) in normal rats. Whether the converted products could induce arrhythmias at the same dose can directly reflect the structural changes in the prototype compound on its cardiotoxicity.

### 2.7. Aconitine-Induced Arrhythmia Test

To further investigate the anti-arrhythmic activity of the converted alkaloids, the testing was performed *in vivo* in 197 rats. The rats were randomly divided into the following 14 groups, and the grouping is shown in Table 1. The blank solvent was configured by taking 4 mL of 1% HCl ethanol and adding saline for volume fixation to 100 mL, the preparation methods of aconitine, positive drugs, and the experimental compounds were the same as the blank solvent. To confirm whether the blank solvent would influence the ECGs of rats, the rats of the blank solvent group were only administered the same volume of blank solvent to observe ECG changes during the recording time.

The rats were anesthetized using 20% urethane (1.2 g/kg, *i.p.*) [19, 20]. Recording the lead II ECGs for 20 min prior to administration, then the experimental compounds, positive drugs, and equal volume of saline were subsequently administered via the exposed vena femoral, respectively. After stabilization for 10 min [21], aconitine was administered into the vena femoral at a dosage of 0.03 mg/kg [22–24] to establish arrhythmia (Figure 1). The onset time of VPB [25] was recorded within 30 min [25–27] after aconitine injection. Meanwhile, the VT [28, 29] or arrhythmia [30], if any, was recorded for each group at the end of observation period.

### 2.8. Statistical Analysis

The data obtained were analyzed using Statistical Package for Social Sciences (SPSS) version 20 software. Experimental data were expressed as mean ± SD or proportion. Descriptive statistics were examined individually. When the data conformed to the normal distribution, the one-way ANOVA would be used for those with homogeneous variance, if the variance was not homogeneous, Tamhane's T2 test would be used for comparison between groups. Comparisons of proportions were made with Pearson's chi-square (*χ*^2^) test. *P* < 0.05 was considered to be a statistically significant difference.

## 3. Results and Discussion

### 3.1. Structural Identification of the Converted Product

Compound **1** was isolated from processed indaconitine by procedures as described in the experimental section.

**Figure 2:**
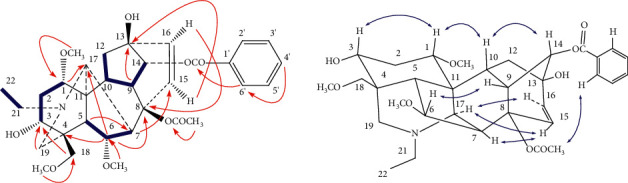
Key ^1^H-^1^H COSY (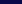), HMBC (H
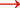
C), and NOESY (H
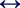
H) correlations of compound **1**.

Δ^15(16)^-16-demethoxyindaconitine (**1**) was obtained as colorless needles, mp 93–95°C, [*α*]_D_^20^ +22.6° (*c* = 1.95, CH_3_OH), and showed a positive reaction with Dragendorff's reagent. Its molecular formula was deduced to be C_33_H_43_NO_9_ from a pseudomolecular ion at *m/z* 598.3004 [M+H]^+^ (calcd. 598.3011) in its HR-ESI-MS. The NMR spectra of compound **1** (Table 2) showed the presence of an *N*-ethyl group (*δ*_H_ 1.05, 3H, t, *J* = 7.32 Hz; *δ*_H_ 2.45, 2.50, each 1H, m; *δ*_C_ 13.3 q, 49.1 t), three methoxyl groups (*δ*_H_ 3.14, 3.23, 3.30, each 3H, s; *δ*_C_ 56.0 q, 57.3 q, 59.2 q), one acetoxyl group (*δ*_H_ 1.33, 3H, s; *δ*_C_ 169.5 s, 21.7 q), a benzoyl group (*δ*_H_ 7.42, 2H, t, *J* = 8.1 Hz; 7.55, 1H, t, *J* = 8.1 Hz; 8.00, 2H, d, *J* = 8.1 Hz; *δ*_C_ 166.8 s, 130.0 s, 129.7 d (2C), 128.4 d (2C), 133.3 d), and four quaternary carbons (*δ*_C_ 43.5, 50.0, 76.1, and 83.7). The aforementioned NMR features suggested an aconitine-type alkaloid for compound **1** [31]. The ^1^H-doublet signal at *δ*_H_ 4.93 (*J* = 5.1 Hz) was assigned to H-14*β* [32, 33], resulting in the location of the benzoyl group to C-14. Three methoxyl groups were attributed to C-1, C-6, and C-18 based on the cross-peaks between 1-OCH_3_ (*δ*_H_ 3.23, s) and C-1 (*δ*_C_ 82.4, d), 6-OCH_3_ (*δ*_H_ 3.14, s) and C-6 (*δ*_C_ 82.3, d), and 18-OCH_3_ (*δ*_H_ 3.30, s) and C-18 (*δ*_C_ 76.9, t) in its HMBC spectrum (Figure 2). Two hydroxyl groups were assigned to C-3 and C-13 based on the correlations between the C-3 (*δ*_C_ 71.7, d) and H-18 (*δ*_H_ 3.68, 3.75), H-19 (*δ*_H_ 2.35, 2.89), as well as C-13 (*δ*_C_ 76.1, s) and H-9 (*δ*_H_ 2.95), H-15 (*δ*_H_ 6.54), in the HMBC of **1**. The ^1^H NMR signal at *δ* 6.54 (1H, d, *J* = 10.26 Hz) and *δ* 6.06 (1H, d, *J* = 10.26 Hz) and the ^13^C NMR signals at *δ* 125.5 and 137.1 indicated the existence of a disubstituted double bond (-CH=CH-) in the molecule. In accordance with the spectral data and the general rules for C_19_-diterpenoid alkaloids, the disubstituted double bond could only be at Δ^2(3)^ or Δ^15(16)^ [34–36]. As mentioned above, a hydroxyl group has already located in the C-3 position. Therefore, the double bond must be at Δ^15(16)^. Also, long-range correlations between C-13 and H-15, and C-8 and H-16 confirmed the presence of a double bond between C-15 and C-16. In addition, the coupling constant between H-15 and H-16 was 10.26 Hz, suggesting that the two hydrogen atoms were CIS structures. The key NOE correlations (Figure 2) between H-15 and H-17, and H-16 and H-17 also confirmed the configuration of H-15 and H-16 in **1** was *α*-orientation. Furthermore, the configuration of 8-OAc in **1** was determined to have a *β*-orientation according to the key NOE correlations between 8-OAc and H-2′, 6′ (Figure 2).

The NMR spectra of compound **1** lacked a methoxyl group at C-16, when compared with indaconitine [34], and the ^13^C NMR spectra of **1** clearly showed changes in the chemical shifts of C-15 and C-16 due to the presence of a double bond. Except for these points, the ^13^C NMR spectra of the two alkaloids were very similar. Thus, the structure of **1** was elucidated as Δ^15(16)^-16-demethoxyindaconitine, a new diterpenoid alkaloid (Figures S1–S14).

### 3.2. Cardiotoxicity of Indaconitine and Its Transformed Product

**Figure 3:**
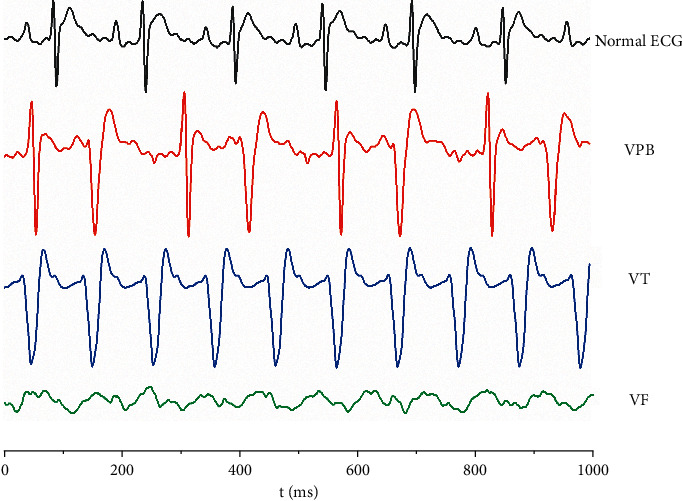
Representative ECGs of SD rats.

Intravenous injection of 0.06 mg/kg indaconitine resulted in disturbances of cardiac rhythm progressing from VPB to VT in all rats, some of the rats developed to VF and even died of fatal ventricular arrhythmias. The incubation period of indaconitine-induced arrhythmia was 148.8 ± 30.7 s. In contrast, no arrhythmias occurred in the Δ^15(16)^-16-demethoxyindaconitine group administering an equal dose. Through this comparative experiment, it could be found that the cardiotoxicity was reduced after processing. The results are shown in Table 3 and Figure 3.

### 3.3. Anti-Arrhythmic Effects of the Converted Products

#### 3.3.1. Effects of the Converted Products on VPB Incubation Period

VPB is the initial manifestation of aconitine-induced arrhythmia model rats, and VPBs are characterized by premature and bizarrely shaped QRS complexes that appear wide on the ECG, these complexes are not preceded by a P wave, and the T wave is usually oriented in a direction opposite the major deflection of the QRS [37, 38]. VPB incubation period refers to the time after the administration of aconitine to the first occurrence of VPB [25]. The length of the VPB incubation period can reflect the anti-arrhythmic effect of the experimental compounds, and the longer the incubation period is, the better the anti-arrhythmic effect is.

The typical characteristic ECGs of VPB and VT occurred successively following the administration of aconitine, even VF occurred in parts of the rats, and arrhythmias lasted for more than 30 min, suggesting that the arrhythmia model was successfully established. The VPB incubation period of the control group was 97.7 ± 23.7 s. Lidocaine and propafenone as the positive anti-arrhythmic drugs delayed the onset time of VPB induced by aconitine in rats, and the latency of VPB was 180.9 ± 51.0 s and 142.3 ± 17.5 s, respectively. Two positive drugs had a significant difference in comparison with the control group (*P* < 0.05). Besides, lidocaine could protect 44.4% of rats from arrhythmia, exhibiting a better anti-arrhythmic effect than propafenone.

**Figure 4:**
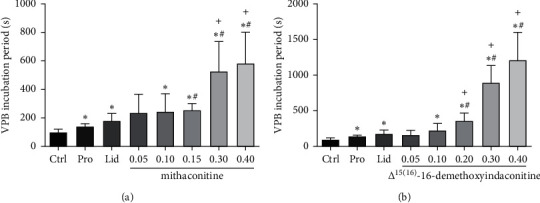
Effects of different experimental compounds on VPB incubation period. Data are presented as mean ± SD. ^∗^*P* < 0.05 compared with the control group, ^+^*P* < 0.05 compared with the lidocaine group, ^#^*P* < 0.05 compared with the propafenone group. (a) Mithaconitine; (b) Δ^15(16)^-16-demethoxyindaconitine.

The effects of mithaconitine and Δ^15(16)^-16-demethoxyindaconitine on VPB incubation period in rats are shown in Table 4 and Figure 4. Compared with the control group, the emergence of VPB in different dose groups of mithaconitine was significantly delayed (*P* < 0.05, Figure 4(a)), except for the 0.05 mg/kg dose group. The VPB incubation periods in 0.15 mg/kg, 0.30 mg/kg, and 0.40 mg/kg groups were 256.6 ± 45.1 s, 527.6 ± 208.6 s, and 583.4 ± 219.7, respectively. They were significantly different from propafenone (*P* < 0.05). Similarly, compared with lidocaine, there was also a significant difference in groups of 0.30 mg/kg and 0.40 mg/kg (*P* < 0.05), indicating that a 0.30 mg/kg or more intravenous dose of mithaconitine exhibited a marked activity relative to the positive drugs.

The double bond of mithaconitine was located at C-8/C-15 rather than C-15/C-16. To exemplify the different positions of the double bonds on the anti-arrhythmic efficacy, the anti-arrhythmic effect of Δ^15(16)^-16-demethoxyindaconitine was further investigated.

In comparison with the control group, different dose groups of Δ^15(16)^-16-demethoxyindaconitine (0.10 mg/kg, 0.20 mg/kg, 0.30 mg/kg, and 0.40 mg/kg) significantly delayed the onset time of VPB (*P* < 0.05; Figure 4(b)). The VPB incubation periods in 0.20 mg/kg, 0.30 mg/kg, and 0.40 mg/kg groups were 363.4 ± 105.0 s, 896.5 ± 239.3 s, and 1211.0 ± 389.1 s, respectively, which were significantly different from propafenone and lidocaine (*P* < 0.05). It was demonstrated that Δ^15(16)^-16-demethoxyindaconitine at the dose range of 0.20–0.40 mg/kg exhibited superior anti-arrhythmic activity relative to mithaconitine and the positive drugs.

In summary, after pretreatment with mithaconitine and Δ^15(16)^-16-demethoxyindaconitine, the onset time of VPB was delayed in all different dose groups. Within their own dose ranges, they could dose-dependently delay the onset time of VPB. Additionally, the VPB incubation periods in 0.30 mg/kg and 0.40 mg/kg dose groups were Δ^15(16)^-16-demethoxyindaconitine > mithaconitine, indicating that Δ^15(16)^-16-demethoxyindaconitine had a better anti-arrhythmic effect than mithaconitine.

#### 3.3.2. Effects of the Converted Products on the Incidence of VT

VT is the result of further development of VPB, a malignant ventricular arrhythmia, as well as an important factor in sudden cardiac death, which can be avoided if VT is corrected promptly and effectively [39]. The incidence of VT can be used to evaluate whether the experimental compounds can effectively prevent the progress of VPB, and the lower the occurrence is, the better the efficacy is.

**Figure 5:**
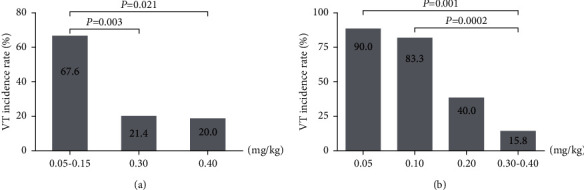
Effect of experimental compounds on the incidence of VT. (a) Mithaconitine; (b) Δ^15(16)^-16-demethoxyindaconitine.

In accordance with the data requirements of the chi-square test, the adjacent dose groups with similar VT incidence should be combined to carry out the chi-square test. Consequently, mithaconitine was divided into three subgroups, 0.05–0.15 mg/kg, 0.30 mg/kg, and 0.40 mg/kg dose groups. As described in Table 5 and Figure 5(a), the chi-square test showed that there was a marked difference in the incidence of VT among the three groups (*χ*^2^ = 12.352, *P*=0.002). In comparison with the 0.05–0.15 mg/kg (67.6%) group, the incidence of VT in 0.30 mg/kg and 0.40 mg/kg groups was significantly dropped to 21.4% and 20.0%, respectively (*χ*^2^ = 8.533, *P*=0.003; *χ*^2^ = 5.340, *P*=0.021).

The subgroups of Δ^15(16)^-16-demethoxyindaconitine were 0.05 mg/kg, 0.10 mg/kg, 0.20 mg/kg, and 0.30–0.40 mg/kg. As displayed in Table 5 and Figure 5(b), the chi-square test showed that there was a significant difference in the incidence of VT among the four groups (*χ*^2^ = 23.332, *P*=0.00003). Compared with the 0.05 mg/kg (90.0%) and 0.10 mg/kg (83.3%) dose groups, the incidence of VT was significantly declined to 15.8% in 0.30–0.40 mg/kg group (*χ*^2^ = 11.973, *P*=0.001; *χ*^2^ = 13.780, *P*=0.0002).

On the basis of the aforementioned results, it can be concluded that mithaconitine and Δ^15(16)^-16-demethoxyindaconitine reduced the incidence of VT in a dose-dependent manner, suggesting that they could effectively prevent the further development of VPB.

#### 3.3.3. Effects of the Converted Products on Arrhythmia Inhibition Rate

The incidence of arrhythmia [30] is defined as the proportion of arrhythmia that occurs within 30 min after pre-intravenous injections of the test compounds, and an arrhythmia model was established with aconitine. The occurrence of any kind of ECGs should be judged as arrhythmias, such as VPB, VT, or VF. The arrhythmia inhibition rate (%) = 100%-arrhythmia incidence rate (%), namely the proportion that no arrhythmia occurs within 30 min, which can be used to evaluate whether the experimental compounds could completely inhibit the arrhythmogenic effect of aconitine. The arrhythmia inhibition rate is the most intuitive index reflecting the strength of the drug efficacy, and the higher it gets, the better the efficacy is.

Considering that the number of rats without arrhythmia in 0.05 mg/kg, 0.10 mg/kg, and 0.15 mg/kg dose groups of mithaconitine was small, the chi-square test could not be performed according to the original grouping. Therefore, the chi-square analysis was carried out by combining the above three groups into 0.05–0.15 mg/kg group. Similarly, the 0.05 mg/kg, 0.10 mg/kg, and 0.20 mg/kg dose groups of Δ^15(16)^-16-demethoxyindaconitine were merged to conduct data analysis.

**Figure 6:**
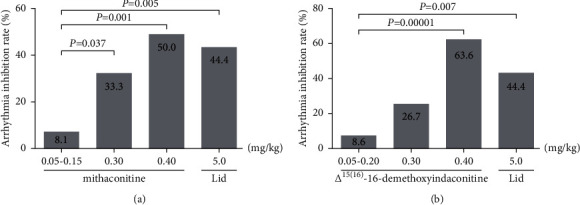
Effect of experimental compounds on arrhythmia inhibition rate. (a) Mithaconitine; (b) Δ^15(16)^-16-demethoxyindaconitine.

In this study, the effects of different doses of mithaconitine (0.05–0.15 mg/kg, 0.30 mg/kg, and 0.40 mg/kg) and lidocaine on the arrhythmia inhibition rate in rats were compared. The chi-square test showed that the arrhythmia inhibition rates in the four groups were significantly different (*χ*^2^ = 14.354, *P*=0.002), as shown in Table 6. Additionally, compared with the 0.05–0.15 mg/kg group, the arrhythmia inhibition rates in 0.30 mg/kg, 0.40 mg/kg, and lidocaine groups were significantly increased from 8.1% to 33.3%, 50.0%, and 44.4%, respectively (*χ*^2^ = 4.337, *P*=0.037; *χ*^2^ = 10.671, *P*=0.001; *χ*^2^ = 7.851, *P*=0.005). Notably, the arrhythmia inhibition rate in the 0.40 mg/kg group was higher than that of lidocaine, exhibiting a better effect, as shown in Figure 6(a).

The chi-square test showed that the arrhythmia inhibition rates in different dose groups (0.05–0.20 mg/kg, 0.30 mg/kg, and 0.40 mg/kg) of Δ^15(16)^-16-demethoxyindaconitine and lidocaine were significantly different (*χ*^2^ = 20.348, *P*=0.0001), as shown in Table 6. Moreover, the arrhythmia inhibition rates of 0.40 mg/kg and lidocaine groups were significantly higher than 0.05–0.20 mg/kg group (*χ*^2^ = 19.571, *P*=0.00001; *χ*^2^ = 7.247, *P*=0.007). Furthermore, the arrhythmia inhibition rate in 0.40 mg/kg group was 63.6%, which was higher than lidocaine (44.4%), demonstrating a better efficacy, as depicted in Figure 6(b).

In summary, within their respective dose ranges, mithaconitine and Δ^15(16)^-16-demethoxyindaconitine could dose-dependently increase the arrhythmia inhibition rate. Furthermore, at the dose of 0.40 mg/kg, the arrhythmia inhibition rates of mithaconitine and Δ^15(16)^-16-demethoxyindaconitine were 50% and 63.6%, respectively, higher than that of the positive drug lidocaine (44.4%), manifesting that these two compounds possessed superior anti-arrhythmic effects.

## 4. Discussion

**Figure 7:**
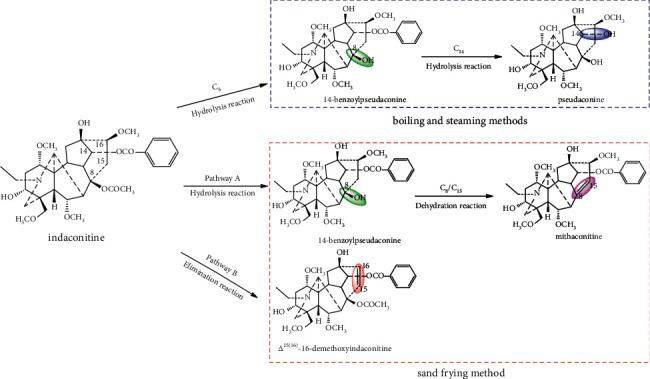
Transformation pathways of indaconitine in the boiling, steaming, and sand frying process.

Currently, *Aconitum* medicinal materials are mainly processed by boiling and steaming in traditional Chinese medicine, and the processing mechanisms underlying the above methods are attributed to diester alkaloids being sequentially converted into less toxic monoester- and amine-alkaloids through ester bond hydrolysis reaction [9, 10]. In our previous study, a similar structural transformation mechanism was found. In the boiling and steaming process, aconitine, deoxyaconitine, and 3-acetylaconitine, the main ingredients of *A. pendulum* Busch, were hydrolyzed to generate benzoylaconine, benzoyldeoxyaconine, and polyschistine-D via hydrolysis of the acetoxyl group at C-8 [11, 40]. Therefore, it could be speculated that indaconitine was sequentially converted into 14-benzoylpseudaconine [41] and pseudoaconine [42] based on the similar transformation mechanism (Figure 7).

In our previous study, mithaconitine, a sand-fried transformed product of indaconitine, has been isolated and identified. It also demonstrated that the cardiotoxicity of mithaconitine was attenuated after processing [12]. In this study, another main transformed product Δ^15(16)^-16-demethoxyindaconitine was further isolated by column chromatography, and the structure was elucidated by NMR and HR-ESI-MS.

By comparing the structural differences between indaconitine and its transformed products, mithaconitine and Δ^15(16)^-16-demethoxyindaconitine, it could be seen that the transformation pathways of indaconitine in the sand frying process were different from that in boiling and steaming process. There might be at least two transformation pathways in the sand frying process: (1) the acetoxyl group at C-8 of indaconitine was firstly hydrolyzed to a hydroxyl group to get 14-benzoylpseudaconine, and then, a double bond at C-8/C-15 was introduced via further dehydration to convert into mithaconitine. (2) Indaconitine was converted into Δ^15(16)^-16-demethoxyindaconitine by elimination of the hydrogen atom at C-15 and the methoxyl group at C-16 (Figure 7).

Thus, the differences between the boiling, steaming, and sand frying methods lay in that, in the first two methods, the acetoxyl group at C-8 of indaconitine was firstly hydrolyzed to a hydroxyl group, and the benzoyl group at C-14 was subsequently hydrolyzed. However, the sand frying method was different. In pathway A, the acetoxyl group at C-8 of indaconitine was firstly hydrolyzed to a hydroxyl group and then formed a double bond at C-8/C-15 through dehydration reaction, but the benzoyl group of C-14 was not changed. In pathway B, the ester bonds at C-8 and C-14 were not changed, but generated a double bond at C-15/C-16. That is, only hydrolysis reaction occurred in the boiling and steaming process. However, not only hydrolysis reactions but also dehydration reactions occurred in the sand frying process.

It is speculated that the reasons for the structural transformation pathways differing among the three processing methods are as follows: (1) the processing temperature is different. The boiling point of water is 100°C under normal atmospheric pressure, and the temperature of water vapor is slightly above 100°C, whereas the gravel can reach 200°C or even higher. (2) The heating medium is different. The medium in contact with the herbs is boiling water or water vapor in the boiling or steaming process, which belongs to the “water and fire processing” [9]. In contrast, the gravel is a solid medium, and there is almost no water involved in the process of sand frying, except for the small amount of moisture contained in the herbs in accordance with the pharmacopoeia, which is more compatible with “fire processing” [43].

The cardiotoxicity test showed that intravenous injection of 0.06 mg/kg indaconitine caused arrhythmias in normal rats, such as VPB, VT, and VF, even 20% of rats died of severe arrhythmias. However, none of the rats in Δ^15(16)^-16-demethoxyindaconitine and mithaconitine [12] groups showed the above arrhythmias. The cardiotoxicity of the transformed products was reduced compared with the prototype compound indaconitine, indicating that the sand frying method contributed to attenuating the cardiotoxicity.

In this study, three indicators, the VPB incubation period, the incidence of VT, and arrhythmia inhibition rate, were used to comprehensively assess the anti-arrhythmic activity of the transformed products of indaconitine. The experimental results manifested that, in the respective dose ranges, mithaconitine and Δ^15(16)^-16-demethoxyindaconitine could dose-dependently delay the VPB incubation period, decline the occurrence of VT, and increase the arrhythmia inhibition rate, indicating that they both had strong anti-arrhythmic activities. For example, mithaconitine could prolong the VPB incubation period in a dose-dependent manner, and the latency of VPB at 0.30 mg/kg and 0.40 mg/kg doses was significantly longer than that of lidocaine and propafenone (*P* < 0.05, Figure 4(a)). Furthermore, the arrhythmia inhibition rate of 0.40 mg/kg group was 50.0%, slightly higher than lidocaine (44.4%) (Figure 6(a)). Similar results were obtained for Δ^15(16)^-16-demethoxyindaconitine. Δ^15(16)^-16-demethoxyindaconitine could dose-dependently delay the onset time of VPB, and the VPB incubation periods were significantly longer than that of propafenone and lidocaine at doses of 0.20 mg/kg, 0.30 mg/kg, and 0.40 mg/kg (*P* < 0.05, Figure 4(b)). The arrhythmia inhibition rate in 0.40 mg/kg group was 63.6%, higher than lidocaine (44.4%) (Figure 6(b)), indicating that its anti-arrhythmic activity was also stronger than lidocaine. Besides, at the optimal dose of 0.40 mg/kg, the incubation periods of mithaconitine and Δ^15(16)^-16-demethoxyindaconitine were 583.4 ± 219.7 s and 1211.0 ± 389.1 s, respectively. Namely, Δ^15(16)^-16-demethoxyindaconitine exhibited a higher arrhythmia inhibition rate and a longer VPB incubation period than mithaconitine, rendering it more promising for development as anti-arrhythmic agents.

## 5. Conclusions

In summary, this study elucidated the structural transformation pathways of indaconitine during the sand frying process by comparing the structures of indaconitine and its transformed products. There might be at least two transformation pathways that exist in the process of sand frying. Besides, cardiotoxicity assays and anti-arrhythmic activity experiments demonstrated that, compared with the prototype compound indaconitine, the two converted products exhibited lower cardiotoxicity and stronger anti-arrhythmic effects, which had the potential to be developed as anti-arrhythmic drugs.

## Figures and Tables

**Table 1:** Grouping of the experimental animals.

No.	Group	Dosage (mg/kg)	Number (n)
1	Blank solvent	—	10
2	Control	—	10
3	Positive	Lidocaine; 5.0 [17]	18
4		Propafenone; 3.2 [18]	9
5	Mithaconitine	0.05	13
6		0.10	14
7		0.15	10
8		0.30	21
9		0.40	20
10	Δ^15(16)^-16-demethoxyindaconitine (**1**)	0.05	10
11		0.10	13
12		0.20	12
13		0.30	15
14		0.40	22

**Table 2:** ^1^H (600 MHz) and ^13^C (150 MHz) NMR data of compound **1** (Δ^15(16)^-16-demethoxyindaconitine).

Compound **1**						Indaconitine
Position	*δ*_H_ (*J* in Hz)	*δ*_C_	HMBC	NOESY	^1^H-^1^H COSY	*δ*_C_
1	3.07 m	82.4 d	C-10, C-17, 1-OCH_3_	H-3, H-10, 1-OCH_3_	H-2*α*, H-2*β*	83.2 d
2*α*	2.32 m	34.1 t	C-4	—	H-1, H-2*β*, H-3*β*	35.1 t
2*β*	2.17 m		C-4	—	H-1, H-2*α*, H-3*β*	
3	3.70 dd (11.7, 4.38)	71.7 d	C-18, C-19	H-1, H-5	H-2*α*, H-2*β*	71.2 d
4	—	43.5 s	—	—	—	43.0 s
5	2.05 d (6.6)	48.2 d	C-7, C-10, C-17, C-18, C-19	H-3, H-18*α*, *β*	H-6	48.6 d
6	4.16 d (6.6)	82.3 d	C-4, C-8, C-17, 6-OCH_3_	H-9, 6-OCH_3_	H-5, H-7	82.0 d
7	2.99 s	45.3 d	C-9, C-11, C-15, C-17	H-15, 6-OCH_3_	H-6	48.6 d
8	—	83.7 s	—	—	—	85.3 s
9	2.95 m	44.1 d	C-12, C-13, C-15	H-6	H-10, H-14	47.2 d
10	2.15 m	41.9 d	C-8, C-17	H-1, H-14	H-9, H-12*α*, *β*	40.7 d
11	—	50.0 s	—	—	—	50.0 s
12*α*	3.02 m	38.8 t	C-11, C-14, C-16	—	H-10, H-12*β*	33.5 t
12*β*	1.89 m		C-9, C-11, C-16	H-14	H-10, H-12*α*	
13	—	76.1 s	—	—	—	74.5 s
14	4.93 d (5.1)	78.1 d	C-8, C-16, ArC=O	H-10, H-12*β*	H-9	78.5 d
15	6.54 d (10.26)	125.5 d	C-9, C-13	H-7, H-17	H-16	39.4 t
16	6.06 d (10.26)	137.1 d	C-8	H-17	H-15	82.8 d
17	3.05 s	62.6 d	C-6, C-8, C-10, C-19	H-15, H-16, H-21, H-22	—	61.4 s
18*α*	3.68 d (9.54)	76.9 t	C-3, C-5, C-19, 18-OCH_3_	H-5, H-19*α*, *β*, 18-OCH_3_	H-18*β*	76.5 t
18*β*	3.75 d (9.54)		C-3, C-5, C-19, 18-OCH_3_	H-5, H-19*α*, 18-OCH_3_	H-18*α*	
19*α*	2.35 d (11.4)	47.4 t	C-3, C-5, C-18, C-21	H-18*α*, *β*	H-19*β*	48.6 t
19*β*	2.89 d (11.4)		C-3, C-17	H-18*α*, H-22	H-19*α*	
21*α*	2.45 m	49.1 t	C-17, C-19	H-17	H-22	47.2 t
21*β*	2.50 m		C-17, C-19	H-17	H-22	
22	1.05 t (7.32)	13.3 q	—	H-17, H-19*β*	H-21*α*,*β*	13.3 q
8-C=O	—	169.5 s	—	—	—	169.2 s
CH_3_	1.33 s	21.7 q	8-C=O	H-2′, 6′	—	21.5 q
1-OCH_3_	3.23 s	56.0 q	C-1	H-1	—	55.5 q
6-OCH_3_	3.14 s	57.3 q	C-6	H-6, H-7	—	57.5 q
18-OCH_3_	3.30 s	59.2 q	C-18	H-18*α*, *β*	—	58.9 q
ArC=O	—	166.8 s	—	—	—	165.7 s
1′	—	130.0 s	—	—	—	129.7 s
2′, 6′	8.00 d (8.1)	129.7 d	C-4′, ArC=O	8-CO-CH_3_	H-3′, 5′	129.2 d
3′, 5′	7.42 t (8.1)	128.4 d	C-1′, C-4′	—	H-2′, 6′	128.1 d
4′	7.55 t (8.1)	133.3 d	C-2′, 6′	—	H-3′, 5′	132.7 d

**Table 3:** Comparison of cardiotoxicity between indaconitine and Δ^15(16)^-16-demethoxyindaconitine (mean ± SD).

Compound	Dose (mg/kg)	VPB incubation period (s)	VT incidence (%)	Mortality (%)
Indaconitine	0.06	148.8 ± 30.7	100	20
Δ^15(16)^-16-demethoxyindaconitine (**1**)	0.06	—	0	0

“—” means that no arrhythmias occurred within 30 min after the injection of the experimental compounds.

**Table 4:** Effects of different compounds on VPB incubation period in aconitine-induced SD rats (mean ± SD).

Compound	Dose (mg/kg)	VPB incubation period(s)
Control	—	97.7 ± 23.7
Lidocaine	5.0	180.9 ± 51.0^*∗*^
Propafenone	3.2	142.3 ± 17.5^*∗*^
Mithaconitine	0.05	237.0 ± 129.8
	0.10	245.2 ± 124.1^*∗*^
	0.15	256.6 ± 45.1^*∗*^^#^
	0.30	527.6 ± 208.6^*∗*^^+#^
	0.40	583.4 ± 219.7^*∗*^^+#^
Δ^15(16)^-16-demethoxyindaconitine	0.05	161.9 ± 63.1
	0.10	224.2 ± 101.6^*∗*^
	0.20	363.4 ± 105.0^*∗*^^+#^
	0.30	896.5 ± 239.3^*∗*^^+#^
	0.40	1211.0 ± 389.1^*∗*^^+#^

^∗^ *P* < 0.05 compared with the control group, ^+^*P* < 0.05 compared with the lidocaine group, ^#^*P* < 0.05 compared with the propafenone group.

**Table 5:** Chi-square analysis of the incidence of VT among different dose groups.

Compound	Dose (mg/kg)	VT (frequency)	Without VT (frequency)	*χ* ^2^	*P*
Mithaconitine	0.05–0.15	23	11	12.352	0.002
	0.30	3	11		
	0.40	2	8		
Δ^15(16)^-16-demethoxyindaconitine	0.05	9	1	23.332	0.00003
	0.10	10	2		
	0.20	4	6		
	0.30–0.40	3	16		

**Table 6:** Chi-square analysis of arrhythmia inhibition rate between different experimental compounds and lidocaine.

Compound	Dose (mg/kg)	Arrhythmia (frequency)	Without arrhythmia (frequency)	*χ* ^2^	*P*
Mithaconitine	0.05–0.15	34	3	14.354	0.002
	0.30	14	7		
	0.40	10	10		
Lidocaine	5.0	10	8		
Δ^15(16)^-16-demethoxyindaconitine	0.05–0.20	32	3	20.348	0.0001
	0.30	11	4		
	0.40	8	14		
Lidocaine	5.0	10	8		

## Data Availability

The data used to support the article are available in the article or supplementary materials.

## Supplementary Materials

Supplementary Materials Figure S1-S13: ^1^H NMR, ^13^C NMR, HSQC, ^1^H-^1^H COSY, HMBC, DEPT, and HR-ESI-MS original spectra of compound **1**. Figure S14: purity test result of compound **1**.

## References

[1] Xiao P.-G., Wang F. P., Gao F., Yan L. P., Chen D. L., Liu Y. (2006). A pharmacophylogenetic study of *Aconitum* L. (Ranunculaceae) from China. *Acta Phytotaxonomica Sinica*.

[2] The Flora Committee of Chinese Academy of Sciences (1979). *Flora of China*.

[3] Ma L., Gu R., Tang L., Chen Z.-E., Di R., Long C. (2015). Important poisonous plants in Tibetan ethnomedicine. *Toxins*.

[4] Nyirimigabo E., Xu Y., Li Y., Wang Y, Agyemang K, Zhang Y (2015). A review on phytochemistry, pharmacology and toxicology studies of *Aconitum*. *Journal of Pharmacy and Pharmacology*.

[5] Editorial Committee of Chinese Pharmacopoeia (2020). *Chinese Pharmacopoeia*.

[6] Wang C.-F., Gerner P., Wang S.-Y., Wang G. K. (2007). Bulleyaconitine A isolated from aconitum plant displays long-acting local anesthetic properties *in vitro* and *in vivo*. *Anesthesiology*.

[7] Li X., Gu L., Yang L., Zhang D., Shen J. (2017). Aconitine: a potential novel treatment for systemic lupus erythematosus. *Journal of Pharmacological Sciences*.

[8] Wang F.-P., Chen Q.-H., Liu X.-Y. (2010). Diterpenoid alkaloids. *Natural Product Reports*.

[9] Liu S., Li F., Li Y., Li W., Xu J., Du H. (2017). A review of traditional and current methods used to potentially reduce toxicity of *Aconitum* roots in Traditional Chinese Medicine. *Journal of Ethnopharmacology*.

[10] Gong Q. F. (2012). *Science of Processing Chinese Materia Medica*.

[11] Wang Y. J., Zhang J., Tian H. P., Zeng C. J., Yao Z., Zhang Y. (2010). Study on the processing principle of *Aconitum* pendulum. *China Journal of Chinese Materia Medica*.

[12] Wang Y., Tao P., Wang Y. J., Deng W. J. (2020). Study on structural transformation pathway of indaconitine in stir-frying with sand process and toxicity of its processing products. *Chinese Traditional and Herbal Drugs*.

[13] Lv G. H., Li Z. B., Yuan L., Chen D. L., Jian X. X., Wang F. P. (1999). Studies on chemical constituents of Kangding monkshood root. *Chinese Traditional and Herbal Drugs*.

[14] Dzhakhangirov F. N., Sultankhodzhaev M. N., Tashkhodzhaev B., Salimov B. T. (1997). Diterpenoid alkaloids as a new class of antiarrhythmic agents. Structure-activity relationship. *Chemistry of Natural Compounds*.

[15] Lin L. Y., Chen Q. H., Wang F. P. (2004). Advances in pharmacological activities of norditerpenoid alkaloids. *West China Journal of Pharmaceutical Sciences*.

[16] Wang F. P. (2009). A deliberation on methodology of modernization of traditional Chinese medicines based on the research and development of new drugs from “Cao Wu”. *Chemical Industry and Engineering Progress*.

[17] Hong B., He J., Le Q., Bai K., Chen Y., Huang W. (2019). Combination formulation of tetrodotoxin and lidocaine as a potential therapy for severe arrhythmias. *Marine Drugs*.

[18] Li H., Niu X., Li G. Z. (2006). Study on effect of Tiaomaiyin injection on experimental arrhythmia. *China Journal of Chinese Materia Medica*.

[19] Bai D.-L., Chen W.-Z., Bo Y.-X. (2012). Discovery of N-(3,5-bis(1-pyrrolidylmethyl)-4-hydroxybenzyl)-4-methoxybenzenesulfamide (sulcardine) as a novel anti-arrhythmic agent. *Acta Pharmacologica Sinica*.

[20] Mohamed O. Y., Al-Masri A. A., El Eter E. A., Lateef R. (2016). SCH. 79797, a selective PAR1 antagonist, protects against ischemia/reperfusion-induced arrhythmias in the rat hearts. *European Review for Medical and Pharmacological Sciences*.

[21] Zhang H. Y., Xu C. Q., Li H. X., Li H. Z., Han L. P., Sun Y. H. (2006). The effect of resveratrol on arrhythmia and cardiac ischemia. *Acta Pharmacologica Sinica*.

[22] Bartosová L., Novák F., Frydrych M. (2005). Effect of a new ultrashort betalytic agent on aconitine-induced arrhythmia. *Biomedical papers of the Medical Faculty of the University Palacky, Olomouc, Czechoslovakia*.

[23] Klekot A. A. (2006). Antiarrhythmic activity of a membrane-protecting agent Sal’magin in rats with aconitine-induced arrhythmias. *Bulletin of Experimental Biology and Medicine*.

[24] Bartosova L., Novak F., Bebarova M. (2007). Antiarrhythmic effect of newly synthesized compound 44Bu on model of aconitine-induced arrhythmia—compared to lidocaine. *European Journal of Pharmacology*.

[25] Sun G. B., Xu H. B., Wen F. C., Ding T., Sun X. B. (2006). Anti-arrhythmic effect of deglucose-chikusetsu-saponin IVa. *Chinese Journal of Pharmacology and Toxicology*.

[26] Zhang J. H., Xiong Y. A. (2015). Effect of paeonol on rats’ ischemic arrhythmia and miRNA-1 expression. *Chinese Journal of Experimental Traditional Medical Formulae*.

[27] Qiu M., Dong Y.-H., Han F. (2016). Influence of total flavonoids derived from *Choerospondias axillaris* foliumon aconitine-induced antiarrhythmic action and hemodynamics in Wistar rats. *Journal of Toxicology and Environmental Health, Part A*.

[28] Meng J. R., Wu T., Huang L. (1992). Influence of neural regulation on anti-arrhythmia effects of GABA in rats. *Acta Pharmacologica Sinica*.

[29] Ma L. Q., Yu Y., Chen H. (2018). Sweroside alleviated aconitine-induced cardiac toxicity in H9c2 cardiomyoblast cell line. *Frontiers in Pharmacology*.

[30] Wang P. D., Ma X. M., Zhang H. L. (1997). Effect of lappaconitine on ECG in anesthetized rats and its anti-arrhythmic action. *Acta Pharmacologica Sinica*.

[31] Pelletier S. W. (1984). *Alkaloids: Chemical and Biological Perspectives*.

[32] Pelletier S. W., Joshi B. S. (1991). Carbon-13 and proton NMR shift assignments and physical constants of norditerpenoid alkaloids. *Alkaloids: Chemical and Perspectives*.

[33] Gao F., Chen D.-L., Wang F.-P. (2006). Two new C_19_-diterpenoid alkaloids from aconitum hemsleyanium var. circinacum. *Chemical and Pharmaceutical Bulletin*.

[34] Pelletier S. W., Mody N. V., Sawhney R. S., Bhattacharyya J. (1977). Application of carbon-13 nmr spectroscopy to the structural elucidation of C_19_-diterpenoid alkaloids from *Aconitum* and *Delphinium* species. *Heterocycles*.

[35] Yue J., Xu J., Chen Y., Chen S. (1994). Diterpenoid alkaloids from *Aconitum* talassicum. *Phytochemistry*.

[36] Desai H. K., Hart B. P., Caldwell R. W., Huang J. H., Pelletier S. W. (1998). Certain norditerpenoid alkaloids and their cardiovascular action. *Journal of Natural Products*.

[37] Hazari M. S., Haykal-Coates N., Winsett D. W., Costa D. L., Farraj A. K. (2009). A single exposure to particulate or gaseous air pollution increases the risk of aconitine-induced cardiac arrhythmia in hypertensive rats. *Toxicological Sciences*.

[38] Casas M. M., Avitia R. L., Gonzalez-Navarro F. F., Cardenas-Haro J. A., Reyna M. A. (2018). Bayesian classification models for premature ventricular contraction detection on ECG traces. *Journal of Healthcare Engineering*.

[39] Wang Q. S., Li Q. G. (2007). Advances in the treatment of ventricular tachycardia. *International Journal of Cardiovascular Disease*.

[40] Wang Y. J., Zeng C. J., Yao Z., Zhang J., Zhang Y., Zhang F. (2010). Diterpene alkaloids from roots and processed products of *Aconitum* pendulum. *Chinese Traditional and Herbal Drugs*.

[41] Khetwal K. S. (2007). Constituents of high altitude himalayan herbs. part 20. a C-19 diterpenoid alkaloid from Aconitum balfourii. *ChemInform*.

[42] Pelletier S. W., Mody N. V., Venkov A. P., Jones S. B. (1979). Alkaloids of delphinium virescens nutt.: virescenine and 14-acetylvirescenine. *Heterocycles*.

[43] Zhong L. Y., Gong Q. F., Yang M., Pan L. (2013). Analysis of feature and development of traditional processing branches. *China Journal of Chinese Materia Medica*.

