# Supplementary material for: Attenuated Structural Transformation of Indaconitine during Sand Frying Process and Anti-Arrhythmic Effects of Its Transformed Products
Source: Evid Based Complement Alternat Med. 2022 Feb 17;2022:8606459. doi: 10.1155/2022/8606459 (PMC8872670; doi:10.1155/2022/8606459)
Supplement: Supplementary Materials — Figure S1-S13: 1H NMR, 13C NMR, HSQC, 1H-1H COSY, HMBC, DEPT, and HR-ESI-MS original spectra of compound 1. Figure S14: purity test result of compound 1. [file 8606459.f1.pdf]

Supplementary Information for

Attenuated Structural Transformation of Indaconitine During Sand Frying Process and Anti-Arrhythmic Effects of Its Transformed Products

Yan Wang, Pei Tao, and Yu-jie Wang

*Yu-jie Wang

E-mail: superwangyj@126.com

This PDF file includes:

Figures S1 to S14

Content

^{1}H NMR of $\Delta^{15(16)}$-16-demethoxyindaconitine 3

^{13}C NMR of $\Delta^{15(16)}$-16-demethoxyindaconitine 5

DEPT of $\Delta^{15(16)}$-16-demethoxyindaconitine 6

Key HSQC correlations of $\Delta^{15(16)}$-16-demethoxyindaconitine 7

Key HMBC correlations of $\Delta^{15(16)}$-16-demethoxyindaconitine 10

Key ^{1}H-^{1}H COSY correlations of $\Delta^{15(16)}$-16-demethoxyindaconitine 12

Key NOESY correlations of $\Delta^{15(16)}$-16-demethoxyindaconitine 13

High-resolution ESI-MS of $\Delta^{15(16)}$-16-demethoxyindaconitine 15

Purity test result of $\Delta^{15(16)}$-16-demethoxyindaconitine 16

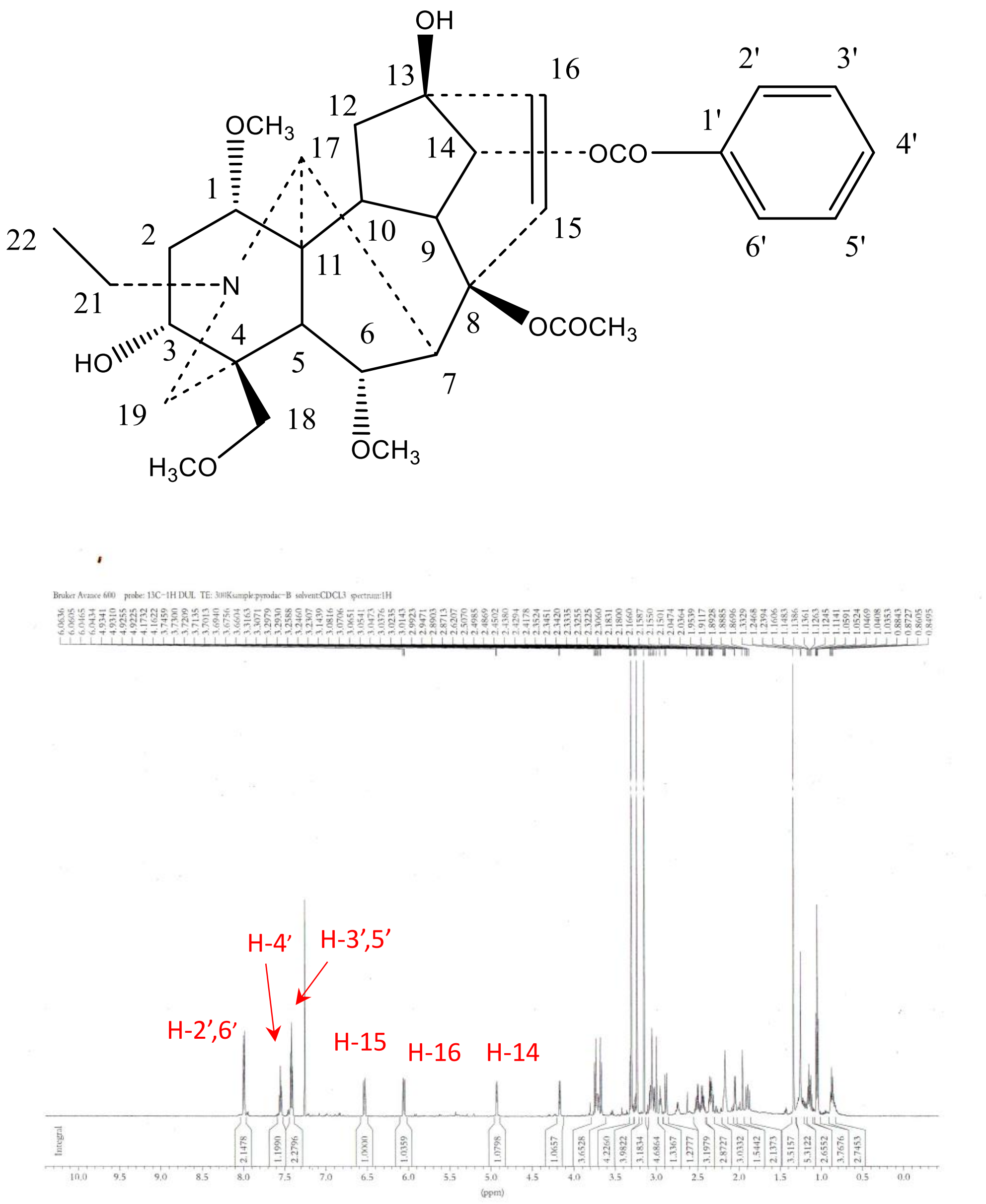

Fig. S1. ^{1}H NMR of $\Delta^{15(16)}$-16-demethoxyindaconitine

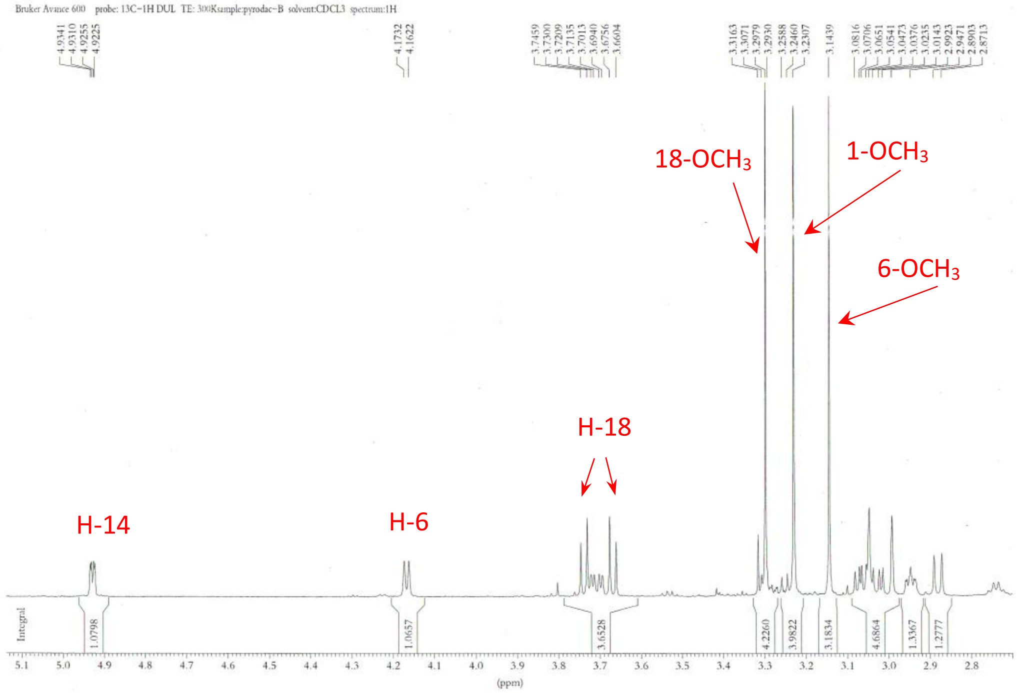

Fig. S2. ^{1}H NMR of $\Delta^{15(16)}$-16-demethoxyindaconitine (Detail)

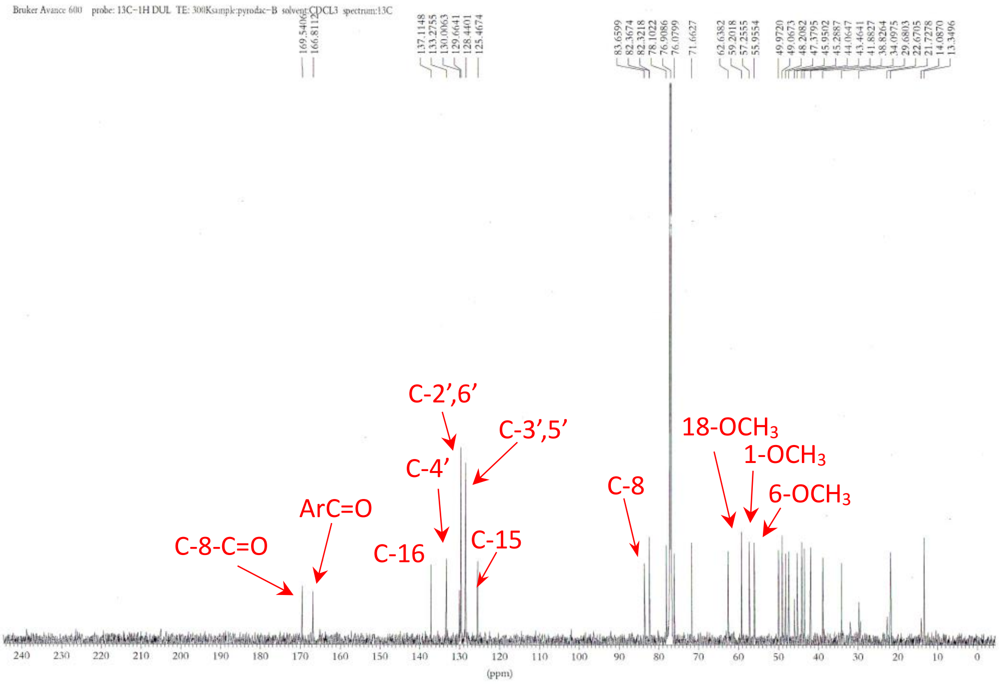

Fig. S3. ^{13}C NMR of $\Delta^{15(16)}$-16-demethoxyindaconitine

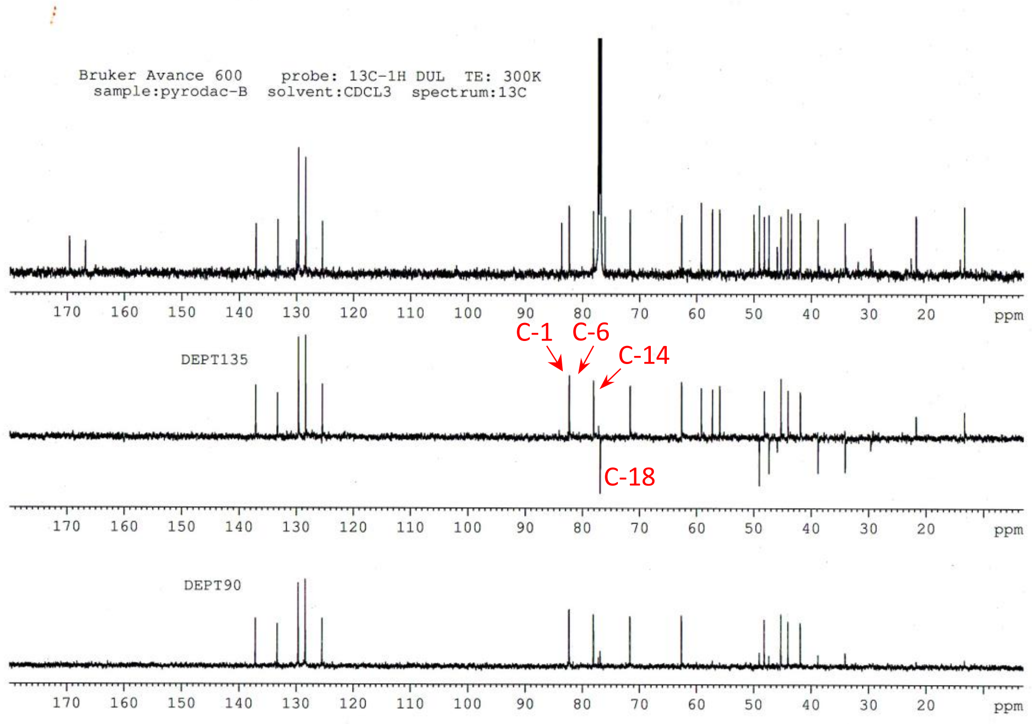

Fig. S4. DEPT of $\Delta^{15(16)}$-16-demethoxyindaconitine

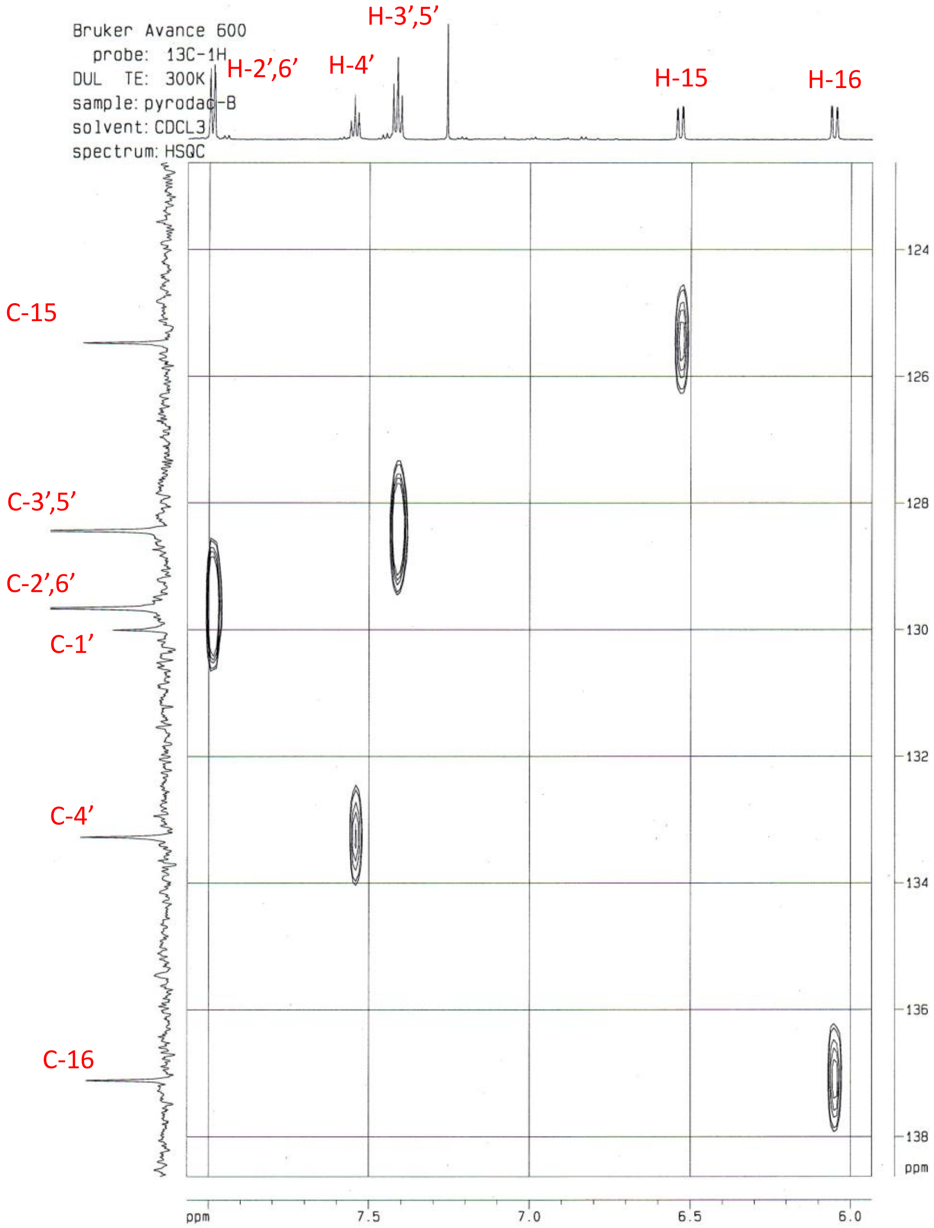

Fig. S5. Key HSQC correlations of $\Delta^{15(16)}$-16-demethoxyindaconitine (Detail 1)

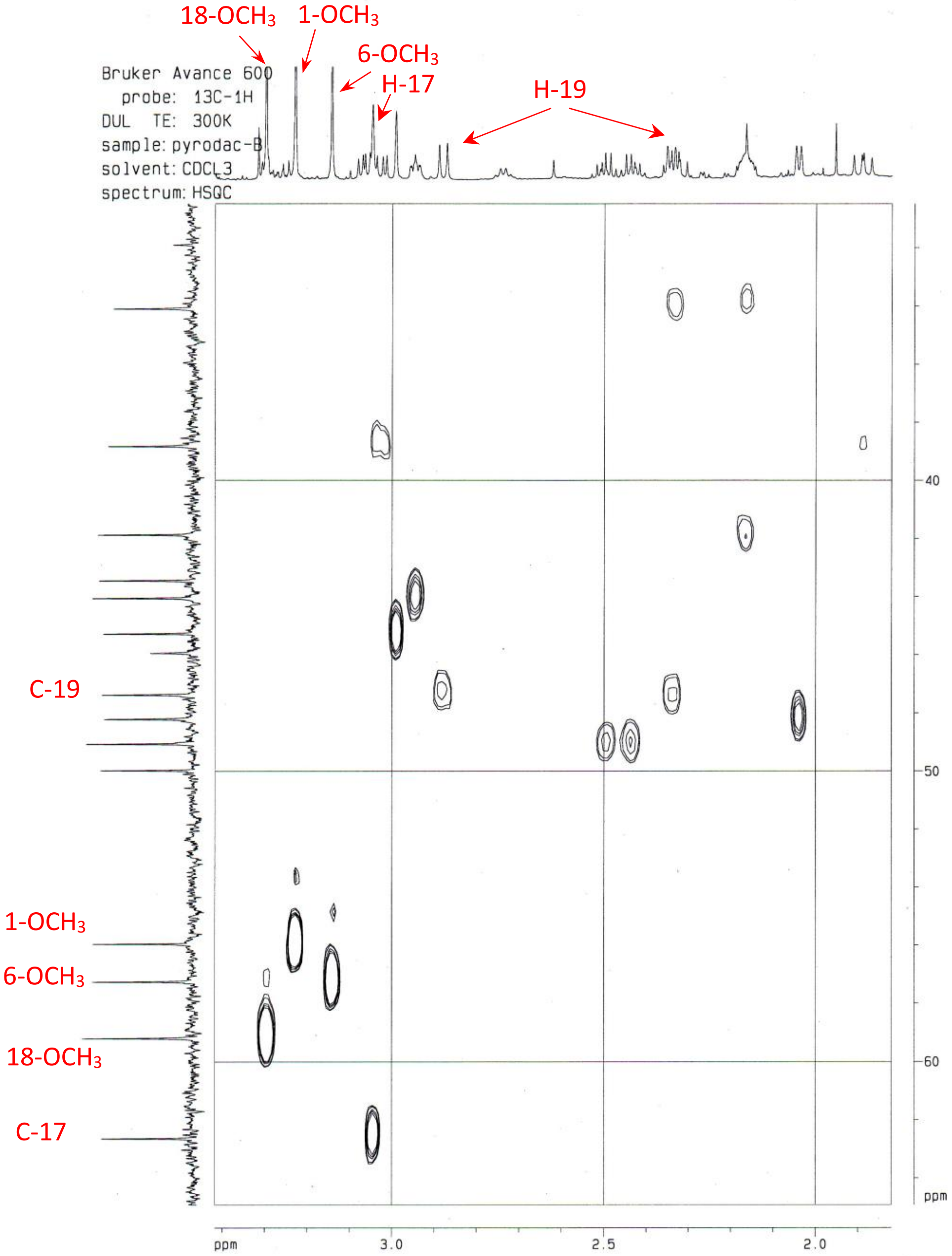

Fig. S6. Key HSQC correlations of $\Delta^{15(16)}$-16-demethoxyindaconitine (Detail 2)

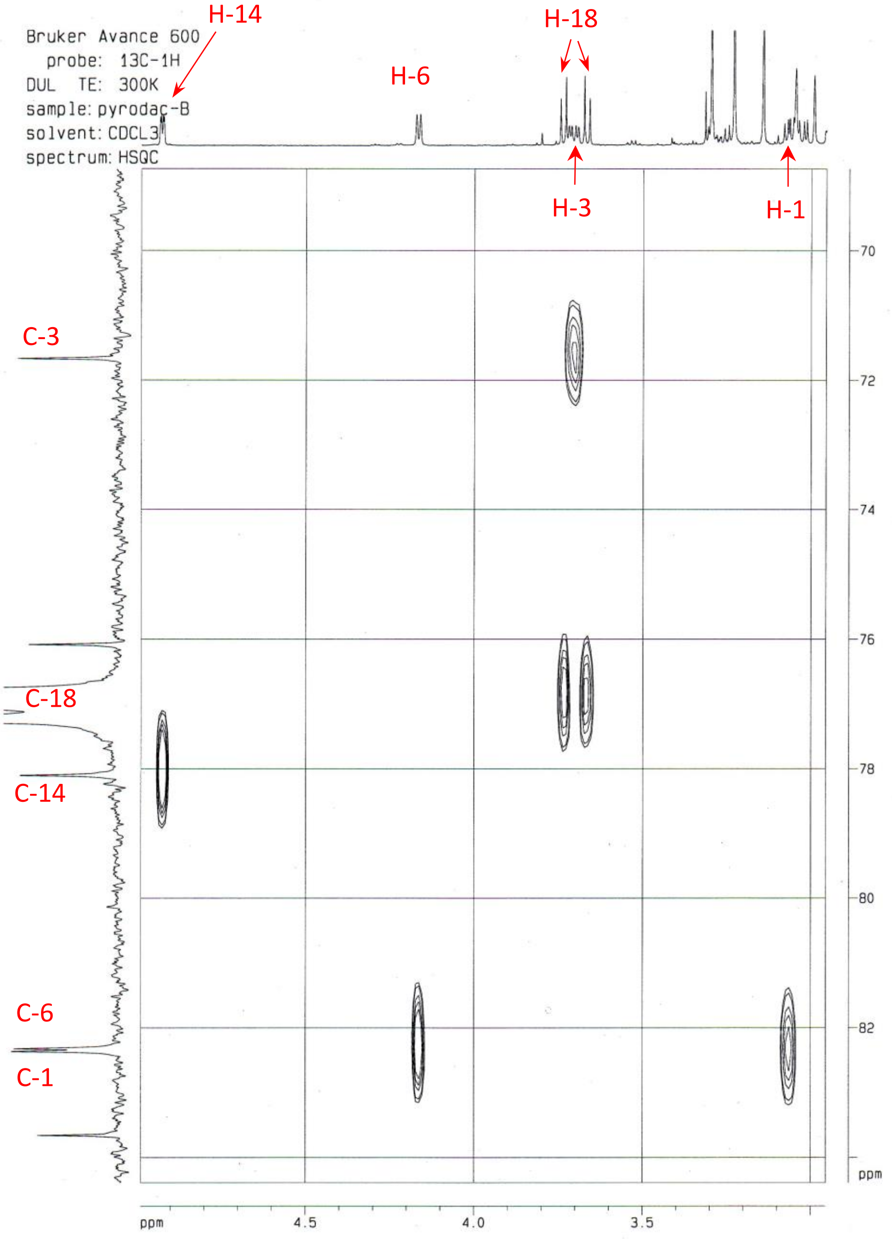

Fig. S7. Key HSQC correlations of $\Delta^{15(16)}$-16-demethoxyindaconitine (Detail 3)

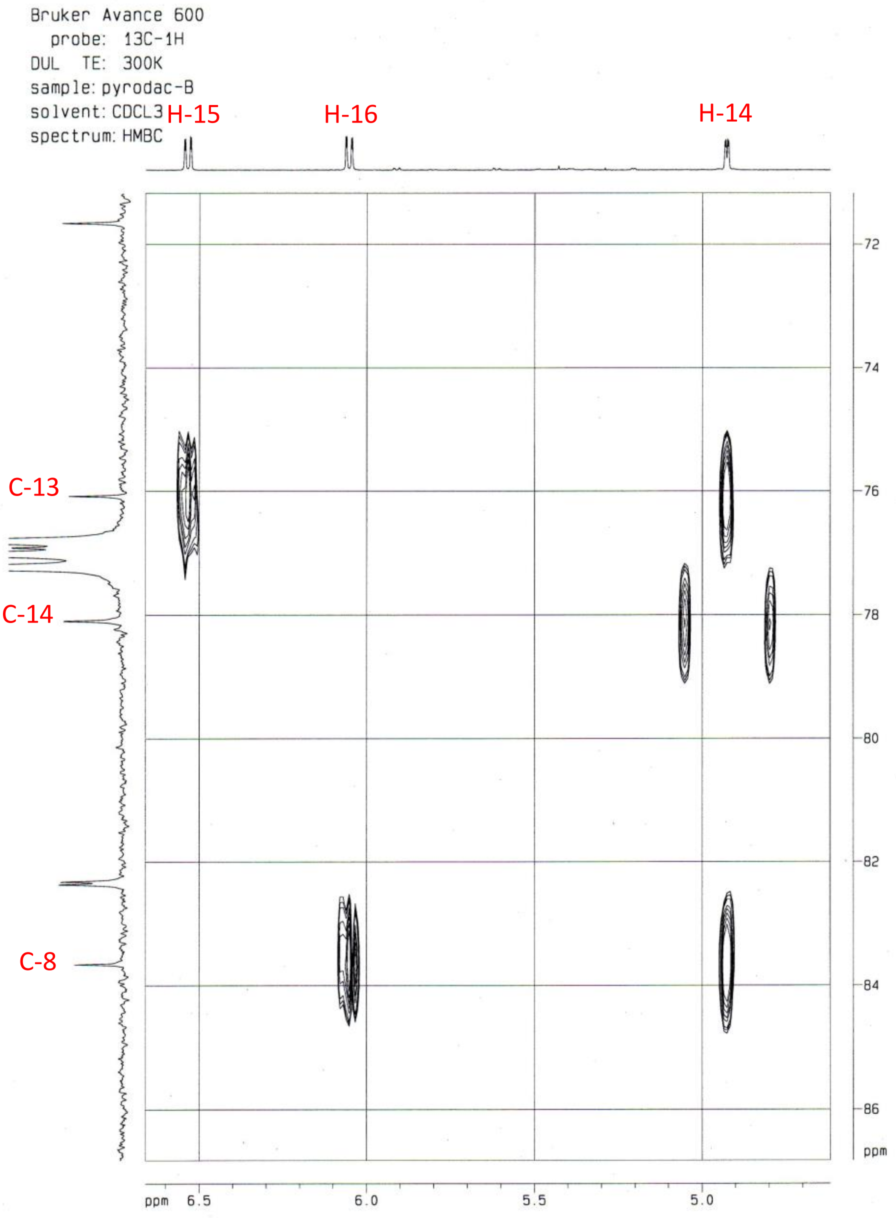

Fig. S8. Key HMBC correlations of $\Delta^{15(16)}$-16-demethoxyindaconitine (Detail 1)

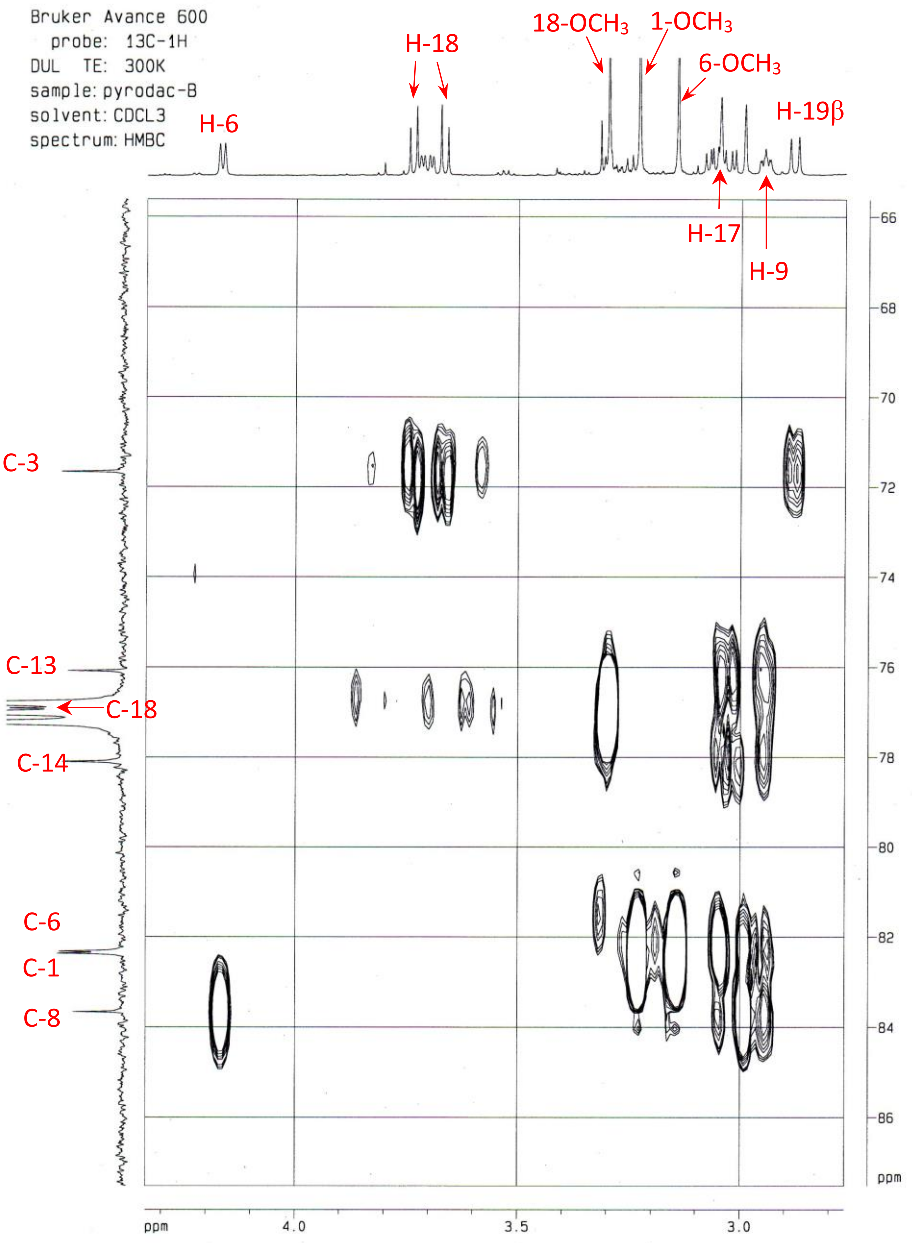

Fig. S9. Key HMBC correlations of $\Delta^{15(16)}$-16-demethoxyindaconitine (Detail 2)

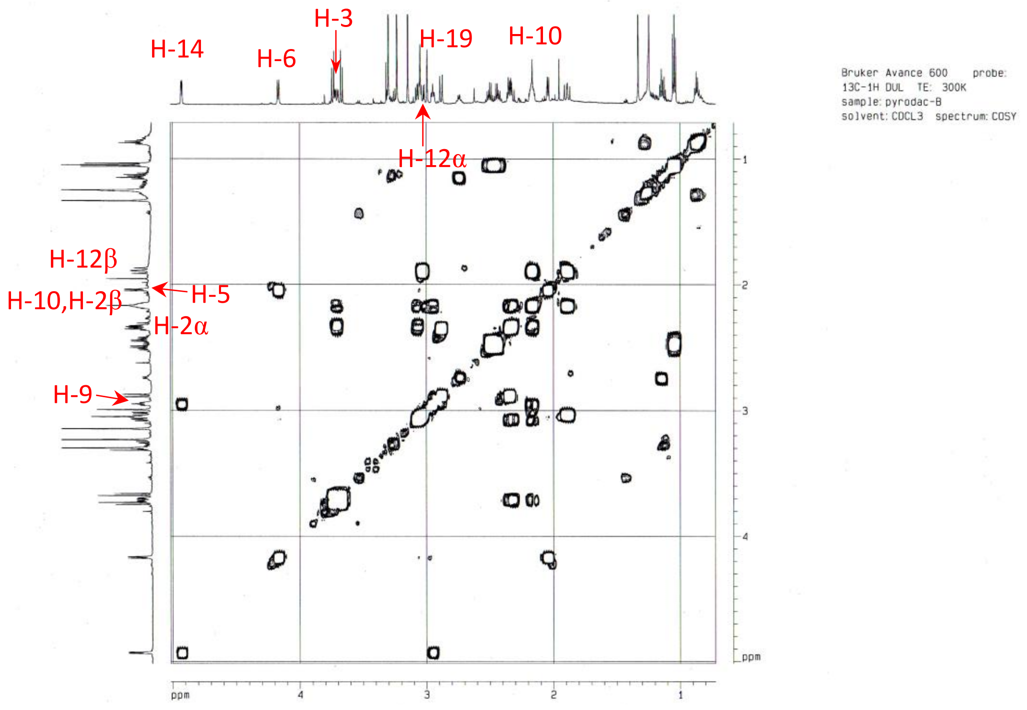

Fig. S10. Key ^{1}H-^{1}H COSY correlations of $\Delta^{15(16)}$-16-demethoxyindaconitine

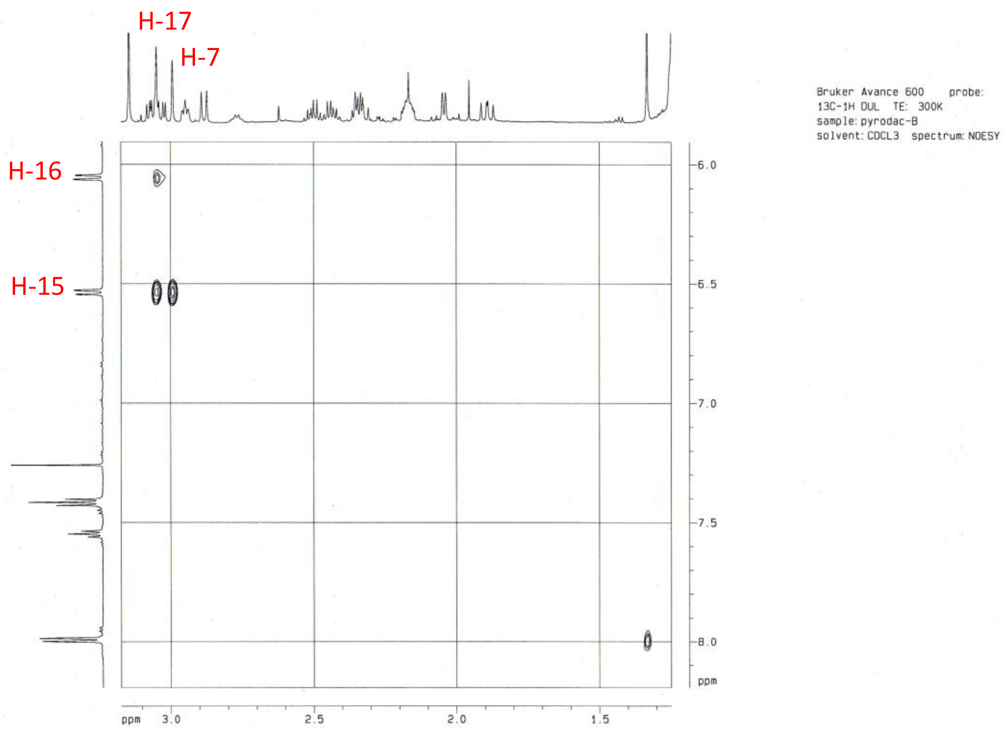

Fig. S11. Key NOESY correlations of $\Delta^{15(16)}$-16-demethoxyindaconitine (Detail 1)

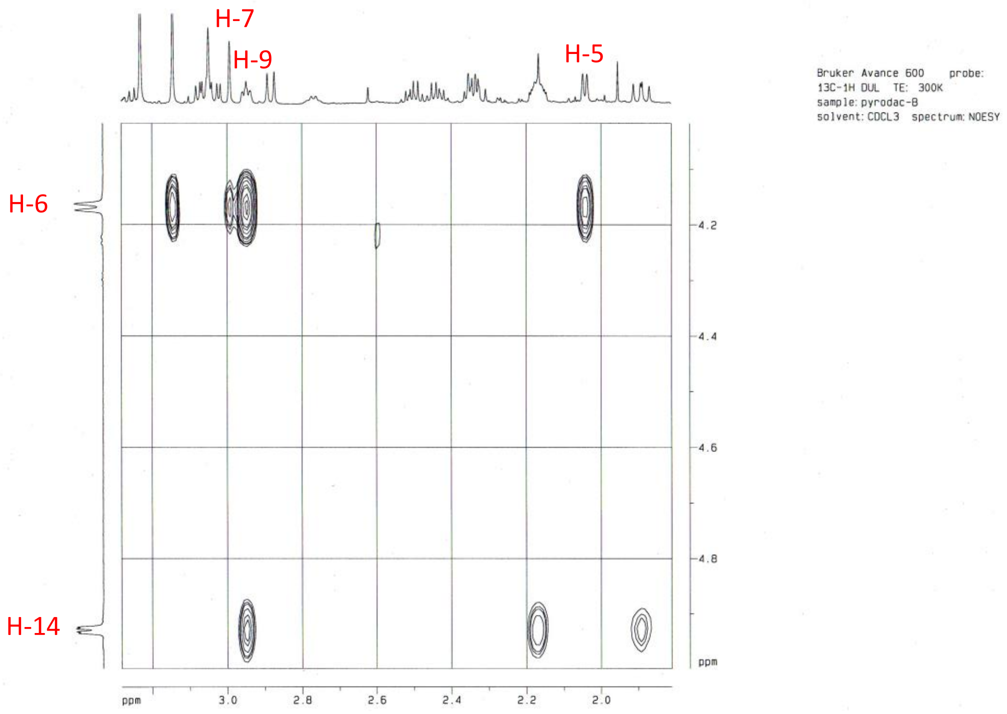

Fig. S12. Key NOESY correlations of $\Delta^{15(16)}$-16-demethoxyindaconitine (Detail 2)

Mass Spectrum SmartFormula Report

Analysis Info

Method	tune_low_NEW.m	Operator	Ma
Sample Name	pyrodac-B	Instrument / Ser#	micrOTOF-Q II 10203
Comment			

Acquisition Parameter

Source Type	ESI	Ion Polarity	Positive	Set Nebulizer	0.8 Bar
Focus	Not active	Set Capillary	4500 V	Set Dry Heater	180 °C
Scan Begin	50 m/z	Set End Plate Offset	-500 V	Set Dry Gas	6.0 l/min
Scan End	1500 m/z	Set Collision Cell RF	150.0 Vpp	Set Divert Valve	Source

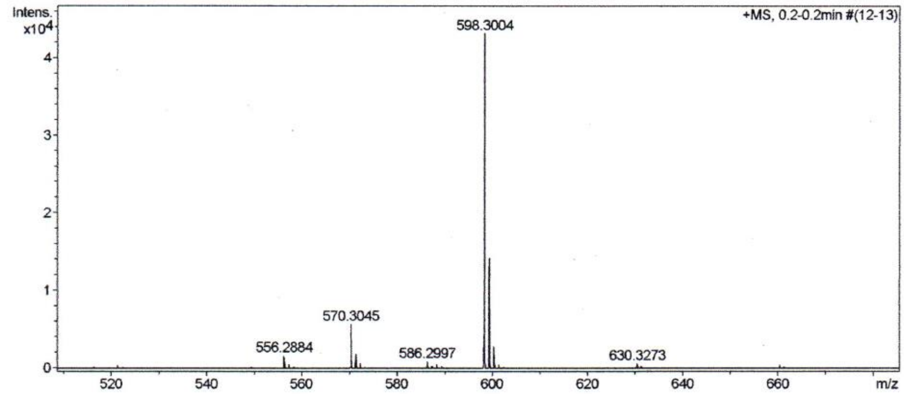

Meas. m/z	Formula	m/z	|err| [ppm]	mSigma	N-Rule	e⁻ Conf
570.3045						
	C 32 H 44 N O 8	570.3061	2.9	21.42	ok	even
598.3004						
	C 31 H 45 N Na O 9	598.2987	3.0	9.33	ok	even
	C 33 H 44 N O 9	598.3011	1.1	20.54	ok	even
	C 32 H 41 N 5 Na O 5	598.3000	0.7	20.92	ok	even
	C 34 H 40 N 5 O 5	598.3024	3.3	32.39	ok	even
	C 37 H 41 N 3 Na O 3	598.3040	6.0	45.31	ok	even
	C 41 H 36 N 5	598.2965	6.5	71.54	ok	even

Bruker Compass DataAnalysis 4.0 Page 1 of 1

Fig. S13. High-resolution ESI-MS of $\Delta^{15(16)}$-16-demethoxyindaconitine

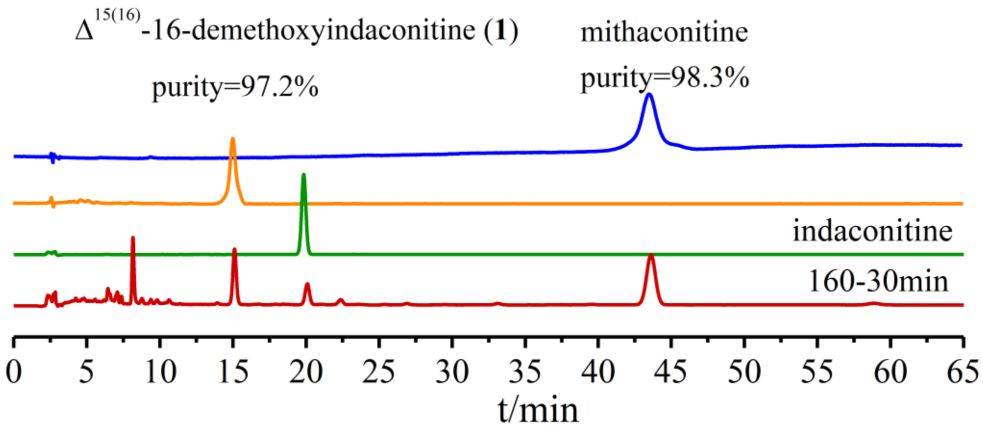

Fig. S14. Purity test result of $\Delta^{15(16)}$-16-demethoxyindaconitine
